# Single and two-dose typhoid conjugate vaccine safety and immunogenicity in HIV-exposed uninfected and HIV-unexposed uninfected Malawian children

**DOI:** 10.1080/21645515.2024.2384760

**Published:** 2024-09-12

**Authors:** Nginache Nampota-Nkomba, Osward M. Nyirenda, Victoria Mapemba, Rhoda Masonga, Priyanka D. Patel, Theresa Misiri, Felistas Mwakiseghile, Richard Wachepa, John M. Ndaferankhande, Bright Lipenga, Pratiksha Patel, Happy Banda, Jennifer Oshinsky, Marcela F. Pasetti, Robert S. Heyderman, Leslie P. Jamka, Divya Hosangadi, Shrimati Datta, Melita A. Gordon, Kathleen M. Neuzil, Matthew B. Laurens

**Affiliations:** a Blantyre Malaria Project, Kamuzu University of Health Sciences, Blantyre, Malawi; b Graduate Program in Life Sciences, University of Maryland School of Medicine, Baltimore, MD, USA; c Malawi-Liverpool-Wellcome Program, Kamuzu University of Health Sciences, Blantyre, Malawi; d Center for Vaccine Development and Global Health, University of Maryland School of Medicine, Baltimore, MD, USA; e Department of Infection, Division of Infectious Diseases, University College London, London, UK; f Malawi-Liverpool-Wellcome Program, University of Liverpool, Liverpool, UK; g Fogarty International Center, National Institute of Health, Bethesda, MD, USA

**Keywords:** Typhoid fever, typhoid vaccine, typhoid conjugate vaccine, HIV-exposed uninfected children, safety, immunogenicity, sub-Saharan Africa

## Abstract

Vaccine safety and immunogenicity data in human immunodeficiency virus (HIV)-exposed uninfected (HEU) children are important for decision-making in HIV and typhoid co-endemic countries. In an open-label study, we recruited Malawian HEU and HIV unexposed uninfected (HUU) infants aged 9 – 11 months. HEU participants were randomized to receive Vi-tetanus toxoid conjugate vaccine (Vi-TT) at 9 months, Vi-TT at 15 months, or Vi-TT at 9 and 15 months. HUU participants received Vi-TT at 9 and 15 months. Safety outcomes included solicited and unsolicited adverse events (AE) and serious AEs (SAEs) within 7 days, 28 days, and 6 months of vaccination, respectively. Serum was collected before and at day 28 after each vaccination to measure anti-Vi IgG antibodies by enzyme-linked immunosorbent assay (ELISA). Cohort 1 (66 participants) enrollment began 02 December 2019, and follow-up was terminated before completion due to the COVID-19 pandemic. Cohort 2 (100 participants) enrollment began 25 March 2020. Solicited AEs were mostly mild, with no significant differences between HEU and HUU participants or one- and two-dose groups. All six SAEs were unrelated to vaccination. Anti-Vi geometric mean titers (GMT) increased significantly from 4.1 to 4.6 ELISA units (EU)/mL at baseline to 2572.0 – 4117.6 EU/mL on day 28 post-vaccination, and similarly between HEU and HUU participants for both one- and two-dose schedules. All participants seroconverted (>4-fold increase in GMT) by the final study visit. Our findings of comparable safety and immunogenicity of Vi-TT in HUU and HEU children support country introductions with single-dose Vi-TT in HIV-endemic countries.

## Introduction

Typhoid fever, an enteric bacterial infection, continues to pose a considerable public health burden that disproportionately affects children in low- and middle-income countries, despite efforts to improve water, sanitation, and hygiene infrastructure, and health education.^1,2^ The rapid emergence and spread of antimicrobial-resistant *Salmonella* Typhi (*S*. Typhi) in recent years has hindered effective typhoid control efforts.^3,4^ In 2018, the World Health Organization (WHO) recommended programmatic use of single-dose typhoid conjugate vaccine (TCV) for children – from 6 months of age – in endemic countries, prioritizing countries with the highest burden of typhoid disease or antimicrobial resistant *S*. Typhi.^5^ To date, three TCVs are WHO pre-qualified. A single dose of TCV is safe, immunogenic, and efficacious in diverse pediatric populations,^6–17^ Effectiveness has also been demonstrated in catch-up campaigns and outbreak settings,^18–21^ including areas with extensively drug-resistant typhoid.^22^

The WHO recommended TCV safety monitoring in special populations, such as immunocompromised individuals, following its TCV vaccination recommendation.^5^ No information is currently available on TCV immunogenicity in sub-Saharan African (SSA) children exposed to human immunodeficiency virus (HIV). Children living with HIV have impaired immune responses to some vaccine antigens, resulting in lower efficacy against disease, leading to recommendations for additional vaccine doses in this population,^23–26^ However, due to increased antiretroviral therapy (ART) availability, mother-to-child HIV transmission is declining, and instead, an increased number of children who are HIV-exposed remain HIV-uninfected (HEU).^27^ In Malawi, prevention of mother-to-child transmission of HIV through universal ART in pregnant women averted of over 7,000 new HIV infections in children in 2022.^28,29^ In 2018, there were approximately 14.8 million HEU children worldwide, comprising more than 15% of annual births in some high-burden countries.^30^ HEU children have higher morbidity and mortality from respiratory and gastrointestinal illnesses than their HUU counterparts.^27,31–33^ As TCV country introductions are considered in SSA countries, where typhoid fever and HIV are co-endemic, TCV safety and immunogenicity in HEU children needs to be established.

This was the first study to investigate TCV safety and typhoid-specific antibody responses to TCV in HEU children. We aimed to 1) determine the safety and immunogenicity of TCV in HEU children, 2) compare responses to single- or two-dose vaccination schedules, and 3) examine whether TCV immune responses are comparable in HEU and HUU children.

## Materials and methods

### Study design and population

In an open label, randomized, safety and immunogenicity study, we recruited HEU and HUU infants aged 9–11 months from Ndirande Health Centre; a government supported primary health-care facility in Blantyre, Malawi. HEU participants were eligible if *in utero* HIV exposure was confirmed by documented maternal history of HIV infection in their mother’s health record. HUU participants were eligible if a negative maternal HIV rapid test (Unigold and Determine) was obtained at enrollment or within 3 months before enrollment. HIV-negative status for HEU participants was confirmed by non-detectable infant HIV viral load on a reverse transcription polymerase chain reaction (PCR) assay at enrollment.

### Ethics statement

The protocol was approved by the Malawi National Health Science Research Committee (Protocol #17/07/11866), the University of Liverpool Research Ethics Committee (Project ID: 2941), and the University of Maryland, Baltimore Institutional Review Board (Protocol #HP-00076625).

### Vaccines and vaccine administration

The study vaccine consists of 25 μg of Vi-antigen polysaccharide conjugated to a nontoxic inactivated tetanus toxoid protein carrier – Vi-TT (Typbar TCV^Ⓡ^, Bharat Biotech International, Hyderabad, India). Vi-TT was administered intramuscularly as a 0.5 mL dose.

HEU participants were randomly assigned at a 1:1:1 ratio into three vaccination groups: Vi-TT at 9 months (HEU9), Vi-TT at 15 months (HEU15), or Vi-TT at 9 and 15 months (HEU9 + 15) of age. All HUU participants were assigned Vi-TT at 9 and 15 months of age.

### Study procedures

We approached parents and guardians of infants attending Ndirande Health Centre, between 9 and 11 months of age and before the first measles-rubella (MR) vaccine administration, to gauge interest; written informed consent was obtained. Children were enrolled at 9–11 months in accordance with the Malawi Expanded Program on Immunization (EPI) schedule for MR and TCV vaccination.^34^ At the 9-month (visit window 9–11 months of age) vaccination visit (V1), length, weight, and mid-upper arm circumference (MUAC) were obtained from eligible participants. HEU9, HEU9 + 15, and HUU groups received Vi-TT in their left thigh, co-administered with MR vaccine in their right thigh, according to the Malawi EPI schedule. Participants in the HEU15 group received MR vaccine only at the 9-month vaccination visit. At the 15-month (visit window 15–18 months of age) vaccination visit (V2), which occurred at least 3 months after the first MR vaccination, participants in the HEU15, HEU9 + 15, and HUU groups received Vi-TT, and all participants received a second MR vaccine per the Malawi EPI. After each vaccination, study clinicians observed participants for 30 min for any immediate adverse events (AEs). In-person or phone follow-up was conducted 7 and 28 days after each vaccination for safety and immunogenicity assessments, respectively. Study procedures are summarized in Supplemental Figure S1 and S2.

### Safety

Safety was measured by comparing the proportion of participants who experienced solicited local and systemic reactions, unsolicited AEs and serious adverse events (SAEs) among the four groups (HEU9, HEU15, HEU9 + 15, and HUU). Local and systemic reactions were solicited 30 min and 7 days after each vaccination. Unsolicited AEs were documented up to 28 days after each vaccination. SAEs were recorded throughout the study follow-up.

### Immunogenicity

Serum was collected from participants at baseline (before vaccination) on V1, and 28 days (visit window ±5 days) after each vaccination to measure anti-Vi immunoglobulin G (IgG) using VaccZyme Human anti-*S*. Typhi Vi enzyme-linked immunosorbent assay (ELISA) kits (The Binding Site Group, MK091). HUU participants had an additional blood draw before vaccination on V2 to document pre-second dose immunogenicity. We measured and compared anti-Vi geometric mean titers (GMT) among HEU and HUU participants and the four vaccination groups. Geometric mean fold rise in titers (GMFR) and percent seroconversion from baseline were calculated. Seroconversion was defined as a four-fold or more increase in antibody titers from baseline to 28 days after vaccination.

### Statistical analysis

The primary endpoint for this descriptive study was Vi-TT immunogenicity. The sample size (*n* = 25 per group) was chosen to be able to detect a 35% or greater difference between HEU and HUU groups, with a desired power of 80%, a type I error rate of 5% and allowing for 10% loss to follow-up. The HUU cohort was a comparator for the two-dose group, as data with one dose were previously reported.^13^

The safety population included all children who received at least one dose of Vi-TT. To measure safety outcomes, we calculated the proportion of participants in each vaccine group (HEU9, HEU15, HEU9 + 15, and HUU) who experienced solicited local and systemic reactions after each vaccination, as well as unsolicited AEs and SAEs during study follow-up. Proportions were compared using Wilson confidence intervals (CI).

For Vi-TT immunogenicity, we conducted a per-protocol analysis that included participants who had blood collected within the allowable window of ±5 days of day 28 post-V1 and V2. Titers below the lower limit of detection, defined as 7.4 EU/mL for Vi antigen, were replaced by one-half the limit of detection. Log_10_ transformed titers and fold-rise were compared across all four groups using one-way ANOVA at 0 and 28 days after each vaccination and described in terms of sample size, geometric mean, and corresponding 95% CIs. Additionally, log_10_ transformed titers and 95% CIs were assessed graphically on a log_10_ scale. The 95% CIs were calculated using the Clopper–Pearson exact method. Log_10_ transformed titers were compared within vaccine groups using the paired t-test. The proportion of participants that seroconverted was compared using Wilson Cis using SAS software, version 9.4.

## Results

Enrollment for cohort 1 began 02 December 2019 and was paused 25 March 2020 due to the COVID-19 pandemic. None of the cohort 1 participants completed all follow-up visits. Most participants missed their D28V1 immunogenicity blood draws, and all participants missed their V2 vaccination visit and all scheduled visits thereafter (Supplemental Figure S1). Enrollment in cohort 2, which included the HUU group, was conducted from 01 March to 27 August 2021 (Supplemental Figure S2).

### Population characteristics

In cohort 1, we screened 81 infants and 15 failed screening due to failed baseline sample collection. We enrolled 66 participants: 23 HEU9, 21 HEU15, and 22 HEU9 + 15 with 63 included in the per-protocol analysis. Baseline characteristics were similar between the groups (Table 1).

**Table 1.** Baseline characteristics.

	Enrolled (N)	TCV Vaccinated at (N)		Mean Age in months (STD)	Median Age in months (Q1-Q3)	Sex N, % (95% CI)		Mean Length (STD)	Mean Weight (STD)	Mean MUAC (STD)	Had Detectable Titer* N, % (95% CI)
		9mo	15mo			Male	Female				
**Cohort 1**											
HEU9	23	23	0	9.5(0.6)	9.2 (9.1–9.8)	9, 39.1 (19.7, 61.5)	14, 60.9 (38.5, 80.3)	68.4(2.6)	8.0(0.9)	14.3(1.0)	4, 20.0 (5.7, 43.4)
HEU15	21	0	0	9.5(0.4)	9.5 (9.1–9.8)	13, 61.9 (38.4, 81.9)	8, 38.1 (18.1, 61.6)	69.4(2.6)	8.2(1.1)	15.0(1.5)	5, 23.8 (8.2, 47.2)
HEU9 + 15	22	22	0	9.5(0.43)	9.3 (9.1–9.9)	13, 59.1 (36.4, 79.3)	9, 40.9 (20.7, 63.7)	69.6(3.4)	8.2(1.1)	14.5(1.3)	4, 18.2 (5.2, 40.3)
HUU9 + 15	NA	NA		NA	NA	NA		NA	NA	NA	NA
**Cohort 2**											
HEU9	27	27	0	9.6(0.8)	9.2 (9.0–9.8)	10, 37.0 (19.4, 57.6)	17, 63.0 (42.4, 80.6)	69.1(2.5)	8.2(1.0)	14.5(1.2)	2, 7.4 (1.0, 24.3)
HEU15	22	0	22	9.5(0.5)	9.4 (9.1–9.7)	13, 59.1 (36.4, 79.3)	9, 40.9 (20.7, 63.7)	69.9(3.2)	8.4(1.1)	14.4(0.9)	3, 13.6 (2.9, 34.9)
HEU9 + 15	26	26	24	9.6 (0.7)	9.4 (9.1–9.8)	12, 46.2 (26.6, 66.6)	14, 53.9 (33.4,73.4)	69.1(3.3)	8.0(1.2)	14.8(1.3)	3, 11.5 (2.5, 30.2)
HUU9 + 15	25	25	24	9.8(0.7)	9.6 (9.3–10.0)	16, 64.0 (42.5, 82.0)	9, 36.0 (18.0, 57.5)	71.3(2.8)	9.1(1.4)	15.3(1.4)	2, 8.0 (1.0, 26.0)

*Detectable titers defined as having ≥7.4 U/mL IgG, detected using S. Typhi Vi VaccZyme IgG EIA kit. TCV: typhoid conjugate vaccine. MUAC = mean upper arm circumference. N = number of participants. CI = confidence interval. STD: standard deviation. Q1-Q3: quartile 1-quartile 3. HEU = HIV exposed, uninfected. HUU = HIV unexposed, uninfected. NA = not applicable. Number enrolled, vaccinated and sex in the intention to treat population. Age, length, height, MUAC, and detectable titers analyzed in the per protocol population. *All cohort 1 HEU15 and HEU9 + 15 participants did not reach V2 due to COVID-19 pause and did not receive TCV.

A total of 109 participants were screened in cohort 2, with 9 failed screenings due to failed sample collection and 100 enrolled: 27 HEU9, 22 HEU15, 26 HEU9 + 15, and 25 HUU. All cohort 2 participants were included in the per-protocol analysis. Mean age and sex distributions did not differ significantly by vaccination group at baseline. However, children in the HUU group had higher mean length, weight, and MUAC than the HEU groups (Table 1).

### Safety

#### Local reactogenicity

In cohort 1, 2 HEU9 + 15 participants experienced local reactions, with 1 reporting mild pain/tenderness and 1 other mild swelling between days 0 and 7 post-V1. No HEU9 participant reported any local reactions. For cohort 2, injection site reactions were mostly mild and occurred in 8/53 (15.1%, 95% CI 7.9–27.1) HEU (HEU9 and HEU9 + 15) and 2/25 (8.0%, 95% CI 2.2–25.0) HUU participants post-V1. Mild or moderate pain/tenderness was the most reported injection site reaction in both groups. Mild erythema and swelling were each reported in one HEU9 participant. By day 7 post-V1, all solicited injection site reactions had been resolved (Table 2).

**Table 2.** Summary of reactogenicity and safety parameters (adverse events) by vaccine group in the ITT population after 9-11-month TCV vaccination.

	Local Reactions at Injection Site, Days 0-7				Systemic Reactions, Days 0-7		
	Pain/Tenderness	Swelling	Erythema	Any Local Reaction	Fever	Irritability	Any Systemic Reaction
**COHORT 1**							
HEU9 (*n* = 22)	0, 0 (0.0–15.4)	0, 0 (0.0–15.4)	0, 0 (0.0–15.4)	0.0 (0.0–15.4)	7, 31.8 (16.4, 52.7)	3, 13.6 (4.8, 33.3) Mild	7, 31.8 (16.4, 52.7)
HEU15 (*n* = 21)	NA	NA	NA	NA	NA	NA	NA
HEU9 + 15 (*n* = 22)	1, 4.6 (0.8, 2.2) Mild	1, 4.6 (0.8, 2.2) Mild	0, 0 (0.0–15.4)	2, 9.1 (2.5, 2.8)	5, 22.7 (10.1, 43.4)	3, 13.6 (4.8, 33.3) Mild 2, moderate 1	7, 31.8 (16.4, 52.7)
HEU9 and HEU9 + 15 (*n* = 44)	1, 2.3 (0.4, 11.8) Mild	1, 2.3 (0.4, 11.8) Mild	0, 0 (0.0–8.0)	2, 4.6 (1.3–15.1)	12, 27.3 (16.4, 41.9)	6, 13.6 (6.4, 26.7) Mild 5, moderate 1	14, 31.8 (20.0, 46.7)
HUU9 + 15 (*n* = 0)	NA	NA	NA	NA	NA	NA	NA
**COHORT 2**							
HEU9 (*n* = 27)	4, 14.8 (5.9, 32.5) Mild	1, 3.7 (0.7, 18.3) Mild	1, 3.7 (0.7, 18.3) Mild	4, 14.8 (5.9, 32.5)	8, 29.6 (15.9, 48.5)	5, 18.5 (8.2, 36.7) Mild 4, Moderate 1	11, 40.7 (24.5, 59.3)
HEU15 (*n* = 22)	NA	NA	NA	NA	NA	NA	NA
HEU9 + 15 (*n* = 26)	4, 15.4 (6.2, 33.5) Mild 3, moderate 1	0, 0 (0.0–13.2)	0, 0 (0.0–13.2)	4, 15.4 (6.2, 33.5)	6, 23.1 (11.0, 42.1)	9, 34.6 (19.4, 53.8) Mild	13, 50 (32.1, 67.9)
HEU9 and HEU9 + 15 (*n* = 53)	8, 15.1 (7.9. 27.1) Mild 7, Moderate 1	1, 1.9 (0.3, 9.9) Mild	1, 1.9 (0.3, 9.9) Mild	8, 15.1 (7.9, 27.1)	14, 26.4 (16.4, 39.6)	14, 26.4 (16.4, 39.6) Mild 13, Moderate 1	24, 45.3 (32.7, 58.6)
HUU9 + 15 (*n* = 25)	2, 8.0 (2.2, 25.0) Mild	0, 0 (0.0–13.7)	0, 0 (0.0–13.7)	2, 8.0 (2.2, 25.0)	3, 12.0 (4.2, 30.0)	4, 16.0 (6.4, 34.7) Mild	7, 28.0 (14.3, 47.6)

Data are n, % (95%CI). n = number of participants. CI = confidence interval. NA = not applicable. HEU = HIV exposed, uninfected. HUU = HIV unexposed uninfected. Cohort 1 did not have a HUU9 + 15 group.

After V2, cohort 2 participants experienced local reactions at similar rates in 5/22 (22.7%, 95% CI 10.1–43.4) first dose HEU15 participants; 4/14 (16.7%, 95% CI 6.7–35.9) second-dose HEU9 + 15 participants; and 5/24 (20.8%, 95% CI 9.2–40.5) second dose HUU participants. Mild and moderate pain/tenderness was reported more frequently than after V1. Erythema was reported in one HEU15 participant. All the local reactions were resolved by day 7 post-V2 (Table 3).

**Table 3.** Summary of reactogenicity and safety parameters (adverse events) by vaccine group in the ITT population after 15-month TCV vaccination.

	Local Reactions at Injection Site, Days 0-7				Systemic Reactions, Days 0-7		
	Pain/Tenderness	Swelling	Erythema	Any Local Reaction	Fever	Irritability	Any Systemic Reaction
**COHORT 2**							
HEU9 (*n* = 27)	NA	NA	NA	NA	NA	NA	NA
HEU15 (*n* = 22)	5, 22.7 (10.1, 43.4) Mild 3, moderate 2	0, 0 (0.0–15.4)	1, 4.5 (0.8, 21.8) Mild	5, 22.7 (10.1, 43.4)	5, 22.7 (10.1, 43.4)	3, 13.6 (4.8, 33.3) Mild 2, moderate 1	7, 31.8 (16.7, 52.7)
HEU9 + 15 (*n* = 24)	4, 16.7 (6.7, 35.9) Mild	0, 0 (0.0–14.3)	0, 0 (0.0–14.3)	4, 16.7 (6.7, 35.9)	7, 29.2 (14.9, 49.2)	5, 20.8 (9.2, 40.5) Mild 3, moderate 2	9, 37.5 (21.2, 57.3)
HEU15 and HEU9 + 15 (*n* = 46)	9, 19.6 (10.7, 33.2) Mild 7, moderate 2	0, 0 (0, 0–7.7)	1, 2.2 (0.4, 11.3) Mild	9, 19.6 (10.7, 33.2)	12, 26.1 (15.6, 40.3)	8, 17.39 (9.1, 30.7) Mild 5, moderate 3	16, 34.8 (22.7, 49.2)
HUU9 + 15 (*n* = 24)	5, 20.8 (9.2, 40.5) Mild 3, moderate 2	0, 0 (0.0–14.3)	0, 0 (0.0–14.3)	5, 20.8 (9.2, 40.5)	7, 29.2 (14.9, 49.2)	4, 16.7 (6.7, 35.9) Mild	9, 37.5 (21.2, 57.3)

Data are n, % (95%CI). n = number of participants. CI = confidence interval. NA = not applicable. HEU = HIV exposed, uninfected. HUU = HIV unexposed uninfected.

Cohort 1 did not receive a vaccination at the 15-month visit and is not included in the table.

### Systemic reactogenicity

Systemic reactions were reported in 14/44 (31.8%, 95% CI 20.0–46.7) of cohort 1 HEU (HEU9 and HEU9 + 15) participants between days 0 and 7 post-V1. In cohort 2, systemic AEs after V1 occurred at a similar rate between HEU and HUU groups in 24/53 (45.3%, 95% CI 32.7–58.6) and 7/25 (28.0%, 95% CI 14.3–47.6) participants, respectively (Table 2). Subjective fever was present at day 7 in 10.3% of HEU and 4.0% of HUU participants. Irritability also persisted at day 7 in 1 HEU participant.

Similar proportions of HEU15 (7/22, 31.8%, 95% CI 16.7–52.7), HEU9 + 15 (9/24, 37.5%, 95% CI 21.2–57.3), and HUU (9/24, 37.5%, 95% CI 21.2–57.3) participants reported systemic AEs after V2 in cohort 2 (Table 3). Fever was common following vaccination but is mostly resolved by day 7 post-V2 except in 4.2% of HEU9 + 15 and 8.3% of HUU participants. All irritability during the first six-day post-vaccination was resolved by day 7 post-V2.

### Other adverse events

No immediate reactions were observed in the first 30-min post-vaccination. In cohort 1, unsolicited AEs occurred in 29/45 (64.4%, 95% CI 48.8–78.1) HEU participants who received Vi-TT, and most were mild and moderate. One participant (2.2%, 0.1–11.8) experienced an SAE for an elective syndactyly hospitalization. In cohort 2, 69/100 (69.0%, 95% CI 60.0–77.9) unsolicited AEs occurred; 53/75 (70.7%, 95% CI 59–80.6) in HEU and 16/25 (64%, 95% CI 42.5–82.8) in HUU participants. Five SAEs occurred in five participants: 4/75 (5.3%, 1.5–13.1) were in HEU and 1/25 (4%, 0.1–20.4) was in HUU. These SAEs were all hospitalizations and were due to gastroenteritis with severe dehydration (*n* = 2), foreign body aspiration (*n* = 1), acute respiratory tract infection (*n* = 1), and febrile seizure (*n* = 1). No unsolicited AEs or SAEs were related to vaccination.

### Immunogenicity

#### Geometric mean titers

**Figure 1.**
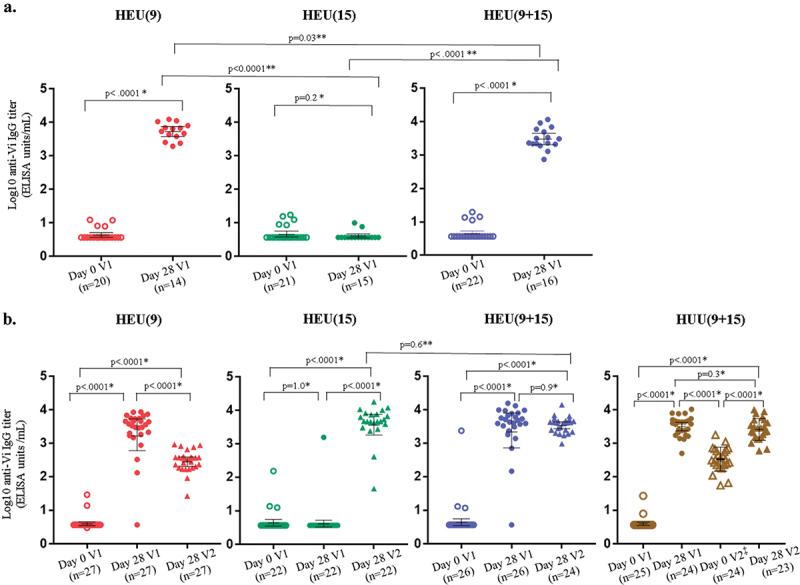
Anti-vi antibody titers. 1A. Cohort 1. 1B. Cohort 2. Anti-vi IgG antibody titers before vaccination (day 0) and 28 days after at 9-month (V1) and 15-month (V2) visit. Colored shapes represent the log_10_ transformed antibody titer result for each individual participant in each corresponding vaccine group. The bar in the middle of individual participant results for each timepoint represents the geometric mean antibody titer value on log_10_ scale with 95% confidence interval. *using the paired t-test on log10 transformed data. **using two sample t-test with unequal variances on log10 transformed data. Using ANOVA on log10 transformed data, HEU(9) at Day 28 V2 and HEU(15) at Day 28 V1 statistically significant difference compared to other 3 groups; *p* < .0001. HEU = HIV exposed, uninfected. HUU = HIV unexposed uninfected. V1: 9-month visit. V2: 15-month visit. ^‡^only HUU (9 + 15) had a blood sample collected on day 0 V2 document pre-second dose immunogenicity.

GMT from baseline to day 28 post-V2 among HEU and HUU participants are shown in Table 3. For cohort 1, anti-Vi GMT rose significantly from day 0 to day 28 among HEU9 and HEU9 +15 participants (Table 4, Figure 1a). In cohort 2, HEU (HEU9 and HEU9 + 15) and HUU participants had similar significant GMT increases from day 0 to day 28 post-V1, reaching 2688.1 EU/mL (95% CI 1713.1–4218.1) and 3493.7 EU/mL (95% CI 2729.4–4471.9), respectively. GMT remained the same between baseline and day 28 post-V1 for HEU-15 participants and were significantly lower than in Vi-TT vaccinated participants at day 28 post-V1 (Table 4, Figure 1b).

**Table 4.** Anti-vi IgG geometric mean titers (GMT) in ELISA units/mL before and 28 days after each vaccination in per-protocol population.

	Baseline V1		Day 28 V1		V2		Day 28 V2	
	N	GMT (95% CI)	N	GMT (95% CI)	N	GMT (95% CI)	N	GMT (95% CI)
**COHORT 1**								
HEU9	20	4.5 (3.7, 5.5)	14	5422.4 (3868.4, 7600.8)	NA	NA	NA	NA
HEU15	21	4.9 (3.8, 6.3)	15	4.2 (3.5, 4.9)	NA	NA	NA	NA
HEU9 + 15	22	4.7 (3.7, 6.0)	16	3108.4 (2114.8, 4568.7)	NA	NA	NA	NA
HEU9 and HEU9 + 15	42	4.6 (4.0, 5.4)	30	4030.0 (3092.2, 5252.3)	NA	NA	NA	NA
HEU15 and HEU9 + 15	-	-	-	-	NA	NA	NA	NA
HUU9 + 15	NA	NA	NA	NA	NA	NA	NA	NA
**COHORT 2**								
HEU9	27	4.2 (3.5, 5.0)	27	2304.6 (1222.2, 4345.4)	NA	NA	27	292.1 (217.5, 392.2)
HEU15	22	4.9 (3.4, 7.2)	22	4.9 (2.8,8.6)	NA	NA	22	4117.6 (2362.8, 7175.8)
HEU9 + 15	26	5.2 (3.1, 8.8)	26	3154.1 (1598.4, 6224.1)	NA	NA	24	3498.7 (2758.1, 4438.0)
HEU9 and HEU9 + 15	53	4.6 (3.6, 6.1)	53	2688.1 (1713.1,4218.1)	-	-	-	-
HEU15 and HEU9 + 15	-	-	-	-	NA	NA	46	3782.1 (2853.0,5013.9)
HUU9 + 15	25	4.1 (3.5, 4.9)	24	3493.7 (2729.4, 4471.9)	24	333.7 (236.1, 471.6)	23	2572.0 (1844.6, 3586.2)

Data are mean (95% CI). N = number of participants. GMT = geometric mean titer. CI = confidence interval. HEU = HIV exposed, uninfected. HUU = HIV unexposed uninfected. NA = not applicable. V1: 9-month visit. V2: 15-month visit. Only Cohort 2 received a vaccination at the 15-month visit and had a HUU group. Only HUU had a blood sample collected on Day 180 to document pre-second dose immunogenicity.

In cohort 2 participants at V2 before vaccination, GMT in the HUU group decreased but remained higher than baseline. Twenty-eight days post-V2, GMT ranged from 2572.0 EU/mL (95% CI 1844.6–3586.2) to 4117.6 EU/mL (95% CI 2362.8–7175.8) and were highest in the HEU15 group that received their first dose of Vi-TT at the V2 visit, followed by the HEU9 + 15 and HUU groups who received their second dose of Vi-TT; these differences were not significant. In HEU9 participants who did not receive a vaccine at V2, GMT decreased but were still higher than baseline. The GMT in the HEU9 group were, however, significantly lower than in the groups that received Vi-TT at V2 (Table 4, Figure 1b).

### Geometric mean fold rise and seroconversion

Table 5 shows GMFR and seroconversion rates from baseline to day 28 post-V1 and 28 days post-V2 among HEU and HUU participants. GMFR from baseline to day 28 post-V1 was high for HEU9 and HEU9 + 15 participants in cohort 1, and all participants seroconverted. In cohort 2, GMFR from day 0 to day 28 post-V1 did not differ between the HEU9, HEU9 + 15, and HUU groups. Conversely, there was a one-fold rise in GMT in HEU15 participants who did not receive Vi-TT. The proportion of V1 vaccinated participants who seroconverted was 94.3% (95% CI 84.3–98.8) in HEU9 and HEU9 + 15, and 100.0% (95% CI 85.8–100.0) in HUU participants. Two of the three HEU participants who did not seroconvert from baseline to day 28 post-V1 were true non-responders, and one had detectable anti-Vi titers at baseline (2367.34 EU/mL). One participant in the HEU15 group seroconverted from baseline to day 28 post-V1, even though HEU15 participants were not assigned to receive Vi-TT at V1.

**Table 5.** Anti-Vi IgG seroconversion and geometric mean fold rise (GMFR) 28 days after each vaccination in per-protocol population.

	From Baseline to Day 28 post-V1				From Baseline to Day 28 post-V2			
	n/N	%Seroconversion (95% CI)	n	GMFR (95% CI)	n/N	% Seroconversion (95% CI)	n	GMFR (95% CI)
**COHORT 1**								
HEU9	14/14	100.0 (76.8, 100.0)	14	1235.1 (791.2, 1928.0)	NA	NA	NA	NA
HEU15	0/15	0.0 (0.0-21.8)	15	0.8 (0.6, 1.1)	NA	NA	NA	NA
HEU9 + 15	16/16	100.0 (79.4, 100.0)	16	597.1 (404.8, 880.8)	NA	NA	NA	NA
HEU9 and HEU9 + 15	30/30	100.0 (88.4, 100.0)	30	838.2 (616.3, 1139.9)	NA	NA	NA	NA
HEU15 and HEU9 + 15	–	–	–	–	NA	NA	NA	NA
HUU9 + 15	NA	NA	NA	NA	NA	NA	NA	NA
**COHORT 2**								
HEU9	26/27	96.3 (81.0, 99.9)	27	553.9 (294.6, 1041.5)	27/27	100.0 (87.2, 100.0)	27	70.2 (49.1, 100.3)
HEU15	1/22	4.6 (0.1, 2.3)	22	1.0 (0.5,2.0)	22/22	100.0 (84.6, 100.0)	22	837.7 (417.4, 1681.0)
HEU9 + 15	24/26	92.3 (74.9, 99.1)	26	606.0 (253.7, 1447.4)	24/24	100.0 (85.8, 100.0)	24	855.5 (649.0, 1127.8)
HEU9 and HEU9 + 15	50/53	94.3 (84.3, 98.8)	53	578.9 (345.4, 970.2)	–	–	–	–
HEU15 and HEU9 + 15	–	–	–	–	46/46	100.0 (92.3, 100.0)	46	846.9 (598.2, 1199.1)
HUU9 + 15	24/24	100.0 (85.8, 100.0)	24	842.7 (634.1, 1119.9)	23/23	100.0 (85.2, 100.0)	23	617.3 (400.6, 951.2)

Seroconversion rates are the percentages of having at least a 4× rise in Anti-Vi IgG titers from day 0. GMFR = geometric mean fold rise in titers from baseline (mean and 95% CI). CI = confidence interval. HEU = HIV exposed, uninfected. HUU = HIV unexposed uninfected. n = number of participants who seroconverted. N = total number in group at timepoint. V1: 9-month visit. V2: 15-month visit. Only Cohort 2 received a vaccination at the 15-month visit and had a HUU group.

From baseline to day 28 post-V2, GMFR among HEU15, HEU9 + 15 and HUU groups in cohort 2 was similar. GMFR at day 28 post-V2 in these Vi-TT recipients was significantly higher than the GMFR of the HEU9 group that did not receive Vi-TT at the V2 visit. GMFR in the HEU9 group at day 28 post-V2 was, however, significantly higher than baseline. By the final visit day, all participants, regardless of the number of Vi-TT doses received or HIV-exposure status, had seroconverted, including two of the three participants who did not seroconvert at day 28 post-V1. One of the three participants who did not seroconvert at day 28 post-V1 and had detectable baseline antibody titers was lost to follow-up before the final blood draw.

## Discussion

In a large randomized controlled efficacy trial in Malawi, a single dose of Vi-TT was efficacious in preventing blood culture-confirmed typhoid fever in children for at least 4 years post-vaccination.^6,17^ Unfortunately, there were too few documented HEU or HIV-infected children to demonstrate efficacy, safety, or immunogenicity in these populations. With this study, we demonstrate that one and two doses of Vi-TT are safe, well tolerated, and immunogenic in Malawian HEU children when co-administered with MR vaccine at the 9- and/or 15-month vaccination visits. Thus, our data are reassuring that a single dose of Vi-TT should be effective in HEU children.

In this study, tolerability, anti-Vi GMT, and seroconversion rates in HEU children do not differ from HUU children and are similar to rates previously reported for HUU Malawian children enrolled in the randomized, controlled efficacy trial,^13^ and other TCV studies.^7–9,11,13,15,35,36^ This is reassuring, as previous studies have shown considerable variation in vaccine responses in HEU children.^37,38^ Studies investigating the safety and immunogenicity of childhood vaccines, including *Haemophilus influenzae* type B (Hib) and pneumococcal conjugate vaccines (PCV) conducted before universal ART for pregnant women showed heterogeneity in antibody titers between HEU and HUU children, but most children attained seroprotective antibody levels.^39–41^ More recent studies conducted in the universal ART era have shown comparable GMTs and seroprotection rates between HEU and HUU children for both protein and conjugate vaccines.^37,42^ Although a few studies report functional impairment in the antibody responses in HEU infants,^43,44^ the majority show equivocal or even increased vaccine responses in HEU children.^45^ Performing efficacy studies may not be feasible in an HEU population, and thus robust immunogenicity, as demonstrated in this cohort, may serve as a marker of efficacy.

Our finding of similar Vi-TT safety and immunogenicity in HEU and HUU children is important programmatically for HIV and typhoid co-endemic countries that have introduced or plan to introduce Vi-TT into routine immunization. In May 2023, Malawi conducted a mass vaccination campaign in children up to 15 years of age, followed by a routine introduction of TCV together with MR vaccine at 9 months of age in the EPI program.^46,47^ Liberia and Zimbabwe have likewise introduced TCV,^48^ and Kenya, Zambia, Burkina Faso, and other SSA countries are in various stages of applying for TCV introduction support from Gavi, the Vaccine Alliance or are preparing for vaccine introduction. Several of these countries also have a high population of HEU infants, as approximately 90% of HEU children reside in SSA.^30^ Routine immunization programs in these countries – and many others on the African continent – follow the same schedule for all children, regardless of HIV status.^26^ The uniformity of safety and immune responses in HEU and HUU children is reassuring and should facilitate integration of TCV into existing immunization programs. All the WHO prequalified TCVs are expected to have robust effectiveness and similar immunogenicity.

Further, our study suggests impaired nutritional status may not impact immune response to Vi-TT. Although the nutritional indicators in the HEU children in our cohort were within normal ranges by international standards,^49^ mean values for length, weight, and MUAC were lower in HEU than HUU children. Still, the two groups amassed a similar immune response and comparable safety profile. Impaired immune responses have been reported in children with poor nutritional status.^50^ It is therefore encouraging that differences in anthropometric measurements did not impact Vi-TT immunogenicity in this study.

Our findings support the WHO-recommended single-dose TCV schedule that has already been implemented in multiple countries in SSA.^5^ In our study, two doses of Vi-TT did not offer better immunogenicity over one dose, although the second dose was well tolerated. Still, a second dose of Vi-TT administered approximately 6 months after the first dose offered some benefit by boosting anti-Vi IgG titers to levels comparable to titers measured after the first dose. There is also the potential for Vi-TT to boost tetanus responses, although this was not tested. Furthermore, co-administration of Vi-TT with MR vaccine has been shown to be safe and produces good immune responses to all three antigens.^13^ It is plausible that preexisting anti-Vi IgG generated by the initial vaccination or exposure to *S*. Typhi may lead to a lower responsiveness to Vi-TT in children if vaccines are given close together. However, it is possible that an additional Vi-TT dose may produce a more robust booster response if administered several years after primary vaccination. A phase 3 Vi-TT trial in India demonstrated a significant booster response when Vi-TT was administered 2 years after primary vaccination, with boosted participants maintaining higher GMT and seroconversion rates compared to un-boosted participants. However, the lack of randomization in the study limits direct comparisons, and further follow-up studies are needed to inform booster-dose immunogenicity.^9,51^

In our study, children who received a first dose of Vi-TT at 9 months had similar immunogenicity and safety profiles compared to children who received their first dose at 15 months. The age of TCV vaccination can therefore be based on country-specific disease epidemiology and EPI schedules. Over 95% of participants seroconverted and seroconversion was sustained for at least 6 months in all children after a single dose of Vi-TT at 9–11 months, which is consistent with the current TCV schedule, at the 9-month vaccination visit in Malawi.

A particular strength of our study is that it was conducted simultaneously with a trial of Vi-TT efficacy in Malawi, including young children exposed to, and living with, HIV.^17^ Therefore, although a correlate of protection has not been established for typhoid fever, Vi-TT efficacy results suggest that a four-fold rise in GMT 1 month after vaccination may be protective.^6,8,14,15^

This study has some limitations. Participants enrolled in cohort 1 did not complete study follow-up due to COVID-19 and had incomplete data. However, retention of cohort 2 participants was strong and provided the data to answer our research questions. While study staff were not blinded to treatment allocation, which may have introduced information bias, the safety and immunogenicity results are consistent with previous data from the Malawi blinded randomized controlled trial.^13^ We did not collect data on the exact duration of solicited local and systemic reactions in this study. However, we reported the status of the reactogenicity at day 7 post-vaccination. Our previous work with Malawian infants captured reactogenicity data with greater granularity and demonstrated the safety of TCV.^13^ We produced short-term immunogenicity data on HEU children vaccinated in their first or second year of life and did not investigate immune response in older HEU children or determine how their immunity wanes over time. We did not test for HIV via PCR in HUU infants and therefore cannot confirm that they were HIV uninfected. However, HIV is rare in infants outside of mother-to-child transmission.^52^ We confirmed that mothers of our HUU participants were HIV uninfected to rule out HIV exposure. Although Vi-TT is conjugated to tetanus, we did not test for anti-tetanus responses in this study, but other studies document a significant anti-tetanus response after Vi-TT vaccination.^7,35^ Despite these limitations, we produced important safety and immunogenicity data for HEU children who will be vaccinated as part of routine immunization programs, which adds value for health policymakers.^10–13^

This study contributes to the growing body of knowledge on Vi-TT safety and immunogenicity and provides new insights into Vi-TT responses in HEU children. Our findings of comparable safety, tolerability, and immunogenicity of Vi-TT in HUU and HEU children provide reassurance to HIV-endemic countries that have introduced single-dose TCV into routine childhood immunization programs and provide data to support new country introductions in similar settings.

## Supplementary Material

Supplemental Material

## Data Availability

After publication, the authors will provide data that underlie the results reported, after de-identification (text, tables, figures), to researchers who provide a methodologically sound proposal with approved aims. Proposals should be directed to mlaurens@som.umaryland.edu; to gain access, data requestors will need to sign a data use agreement.
