# Supplementary material for: Single and two-dose typhoid conjugate vaccine safety and immunogenicity in HIV-exposed uninfected and HIV-unexposed uninfected Malawian children
Source: Hum Vaccin Immunother. 2024 Sep 12;20(1):2384760. doi: 10.1080/21645515.2024.2384760 (PMC11404620; doi:10.1080/21645515.2024.2384760)
Supplement: Supplemental Material [file KHVI_A_2384760_SM9033.pdf]

Vaccine group	HEU9	HEU15	HEU9+15	HUU9+15
**Enrolled**	26	29	26	NA
**TCV vaccinated at 9 months (V1)**	23	NA	22	NA
**Day 0**				
• Serum for immunogenicity assessment	X	X	X	–
• Serum for testing presence of HIV RNA	X	X	X	–
• Vaccination (9-11 months)	√	–	√	–
• Assess and record SAEs	X	X	X	–
• Assess and record solicited AEs	X	X	X	–
**Day 7 (-2/+3 days)**				
**Day 28 (+/- 5 days)**				
• Serum for immunogenicity assessment	X	X	X	–
• Assess and record SAEs	X	X	X	–
• Assess and record solicited AEs	X	X	X	–
**TCV vaccinated at 15 months (V2)***	NA	NA	NA	NA

Supplemental Figure 1. Disposition of participants (CONSORT flow diagram) for Cohort 1.
HEU=HIV exposed, uninfected. HUU=HIV unexposed, uninfected. NA= Not Applicable. *All first cohort HEU15 and HEU9+15 participants did not reach V2 due to COVID-19 pause and did not receive TCV. Cohort 1 did not have a HUU9+15 group.

Vaccine group	HEU9	HEU15	HEU9+15	HUU9+15
**Enrolled**	28	25	27	29
**TCV vaccinated at 9 months (V1)**	27	NA	26	25
**Day 0**				
• Serum for immunogenicity assessment	X	X	X	X
• Serum for testing presence of HIV RNA	X	X	X	–
• Vaccination (9-11 months)	√	–	√	√
• Assess and record SAEs	X	X	X	X
• Assess and record solicited AEs	X	X	X	X
**Day 7 (-2/+3 days)**				
• Assess and record solicited AEs	X	X	X	X
**Day 28 (+/- 5 days)**				
• Serum for immunogenicity assessment	X	X	X	X
• Assess and record SAEs	X	X	X	X
• Assess and record solicited AEs	X	X	X	X
**TCV vaccinated at 15 months (V2)***	NA	22	24	24
**Day 0**				
• Serum for immunogenicity assessment	–	–	–	X
• Vaccination (15-17 months)	–	√	√	√
• Assess and record SAEs	X	X	X	X
• Assess and record solicited AEs	X	X	X	X
**Day 7 (-2/+3 days)**				
• Assess and record solicited AEs	X	X	X	X
**Day 28 (+/- 5 days)**				
• Serum for immunogenicity assessment	X	X	X	X
• Serum for testing presence of HIV RNA	X	X	X	–
• Assess and record SAEs	X	X	–	X
• Assess and record solicited AEs	X	X	–	X

Supplemental Figure 2. Disposition of participants (CONSORT flow diagram) for Cohort 2.
HEU=HIV exposed, uninfected. HUU=HIV unexposed, uninfected.
